# EAAC1 is expressed in rat and human prostate epithelial cells; functions as a high-affinity L-aspartate transporter; and is regulated by prolactin and testosterone

**DOI:** 10.1186/1471-2091-7-10

**Published:** 2006-03-27

**Authors:** Renty B Franklin, Jing Zou, Ziqiang Yu, Les C Costello

**Affiliations:** 1 Department of Biomedical Sciences/Dental School, University of Maryland, Baltimore, Maryland, USA

## Abstract

**Background:**

Prostate epithelial cells accumulate a high level of aspartate that is utilized as a substrate for their unique function of production and secretion of enormously high levels of citrate. In most mammalian cells aspartate is synthesized; and, therefore is a non-essential amino acid. In contrast, in citrate-producing prostate cells, aspartate is an essential amino acid that must be derived from circulation. The prostate intracellular/extracellular conditions present a 40:1 concentration gradient. Therefore, these cells must possess a plasma membrane-associated aspartate uptake transport process to achieve their functional activity. In earlier kinetic studies we identified the existence of a unique Na+-dependent high-affinity L-aspartate transport process in rat prostate secretory epithelial cells. The present report is concerned with the identification of this putative L-aspartate transporter in rat and human prostate cells.

**Results:**

The studies show for the first time that EAAC1 is expressed in normal rat prostate epithelial cells, in normal and hyperplastic human prostate glands, and in human malignant prostate cell lines. EAAC1 expression and high-affinity L-aspartate transport are correspondingly down-regulated by EAAC1 siRNA knock down. Exposure of prostate cells to physiological levels of prolactin or testosterone results in an up-regulation of EAAC1 expression and a corresponding increase in the high-affinity transport of L-aspartate into the cells.

**Conclusion:**

This study shows that EAAC1 functions as the high-affinity L-aspartate transporter that is responsible for the uptake and accumulation of aspartate in prostate cells. In other cells (predominantly excitable tissue cells), EAAC1 has been reported to function as a glutamate transporter rather than as an aspartate transporter. The regulation of EAAC1 expression and L-aspartate transport by testosterone and prolactin is consistent with their regulation of citrate production in prostate cells. The identification of EAAC1 as the high-affinity L-aspartate transporter now permits studies to elucidate the mechanism of hormonal regulation of EAAC1 gene expression, and to investigate the mechanism by which the cellular environment effects the functioning of EAAC1 as an aspartate transporter or as a glutamate transporter.

## Background

Normal prostate secretory epithelial cells have the specialized and unique function of synthesizing and accumulating extraordinarily high levels of citrate for secretion as a major component of prostatic fluid [for recent reviews of prostate citrate metabolism see [1-3]]. This requires a continual availability of carbon sources for the intramitochondrial production of acetyl coenzyme A and oxalacetate for the synthesis of citrate. The former is derived from glucose via pyruvate formation and oxidation, and the latter is derived from aspartate via transamination with glutamate (mitochondrial aspartate aminotransferase reaction; mAAT). In these specialized prostate cells, aspartate is an essential amino acid that is derived from circulation. As represented in rat ventral prostate glandular epithelial cells, the cellular concentration of aspartate is ~1.2 mM [4-6]. The plasma level of aspartate is ~0.03 mM. Therefore the uptake and accumulation of cellular aspartate occurs against a 40:1 concentration gradient. This is achieved by the existence of a Na+-dependent high-affinity L-aspartate transport process with kinetic properties that result in cellular accumulation of aspartate from circulation [5-7]. The kinetic properties are representative of the Na+- dependent high-affinity glutamate-aspartate transporters referred to as the X_AG_^- ^class of amino acid transporters. The general characteristics of this class include: plasma membrane transporters; transport either glutamate or aspartate with high affinity; Na+ coupled transport; no or low affinity for neutral and basic amino acids [8,9].

An important unresolved issue was the identification of the putative prostate high-affinity L-aspartate transporter. Subsequent to our kinetic identification of the transport process, significant advances have been achieved in the genetic and protein identification and characterization of the X_AG_^- ^transporter class as EAATs (excitatory amino acid transporters). This class includes EAAT1 (GLAST1); EAAT2 (GLT1); EAAT3 (EAAC1); EAAT4. The dominance of the reported studies in excitatory cells has resulted in the EAATs being described functionally as glutamate transporters. Nevertheless, we focused on the possibility that a member of this class of transporters, particularly the ubiquitously expressed EAAC1, could be the functional high-affinity L-aspartate transporter in prostate cells. Our earlier studies (6) demonstrated that the high-affinity transport of aspartate is regulated by testosterone, which appeared to be dependent upon its regulation of gene expression of a putative transporter. In this present report we show that EAAC1 (Primary accession number P43005; gene *SLC1A1*) is expressed in normal rat prostate epithelial cells, in human prostate glandular tissue, and in human prostate malignant cell lines; and EAAC1 functions as a high-affinity L-aspartate transporter in rat and human prostate cells; and EAAC1 expression is regulated by testosterone and prolactin.

## Results

**Figure 1:**
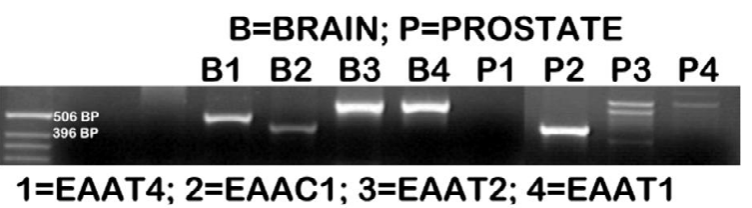
Expression of EAATs (RT-PCR) in rat brain and in rat ventral prostate cells.

In the absence of published information regarding the identification of specific aspartate transporters in prostate, we elected to determine if any members of the EAAT class were expressed in prostate cells. Because we had identified the high-affinity L-aspartate transport process in rat ventral prostate cells [5-7], we first determined the expression (RT-PCR) of EAATs in these cells; and, for comparison, in brain tissue that is known to express these transporters. As shown in figure 1, EAATs 1–4 are expressed in brain tissue as expected. In rat ventral prostate cells: EAAT 4 is not expressed; EAAT 1 and EAAT 2 seem to be weakly expressed. Most importantly, EAAC1 is prominently expressed in the ventral prostate cells. This led us to expect that EAAC1 might be the functional high-affinity L-aspartate transporter in prostate cells.

**Figure 2:**
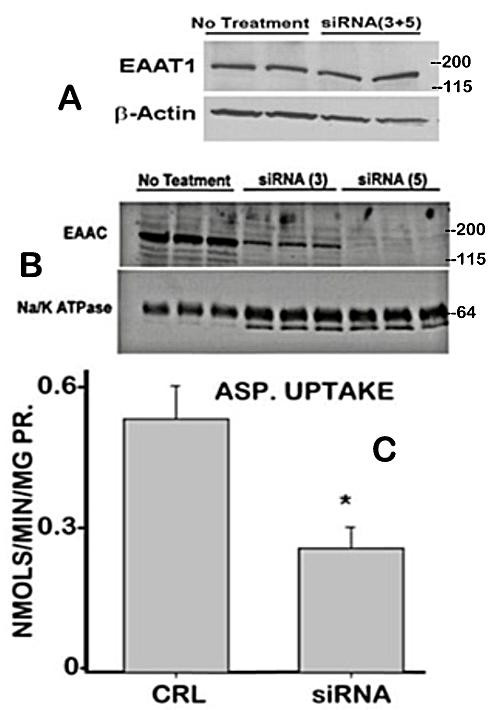
Effects of EAAC1 siRNA on EAAC1 expression and aspartate uptake by PC-3 cells. **A. **The specificity of the EAAC1siRNA is demonstrated by the absence of any effect on EAAT1. **B. **EAAC1 siRNA knocks down the level of EAAC1 in the membrane fraction of PC-3 cells. Na/K ATPase used as a membrane protein for loading control. siRNA(3) and siRNA(5) are 2 siRNA pool preparations based on difference sequences of EAAC1. **C. **Specific knock- down of EAAC1 decreases high-affinity uptake of aspartate by PC-3 cells

The establishment of EAAC1 as the functional high-affinity L-aspartate transporter requires the demonstration that altered expression of EAAC1 results in altered aspartate uptake. In order to determine this relationship we elected to employ the human prostate PC-3 cell line. However, it was necessary to determine if PC-3 cells exhibit aspartate uptake under the conditions representative of the high-affinity L-aspartate transport that we had established in rat ventral prostate cells. As shown in figure 2, PC-3 cells exhibit high-affinity L-aspartate uptake. We also determined if PC-3 cells contained membrane-associated EAAC1, and figure 2 establishes the presence of EAAC1 transporter protein. These relationships in the PC-3 cells made it possible to determine the effect of EAAC1 knock-down on the abundance of EAAC1 transporter and on the high-affinity transport of aspartate. Figure 2 shows that the abundance of EAAC1 transporter is effectively down-regulated in siRNA transfected PC-3 cells. Since siRNA(5) appeared to be more effective than siRNA(3) subsequent experiments were carried siRNA(5). Correspondingly, the results demonstrate that knock-down of EAAC1 markedly decreases the high-affinity uptake of aspartate by PC-3 cells. The specificity of the siRNA for EAAC1 is demonstrated by the absence of knock-down of EAAT1 that is also expressed in these cells. It is evident that some aspartate transport activity remains after siRNA knock-down of EAAC1. This residual activity is likely partly due to some existing low level of EAAC1, and also due to some additional aspartate transport mechanism that we earlier described as low-affinity transport [5]. Nevertheless, the major point is the demonstrated down regulation of EAAC1 along with significant reduction of aspartate transport.

**Figure 3:**
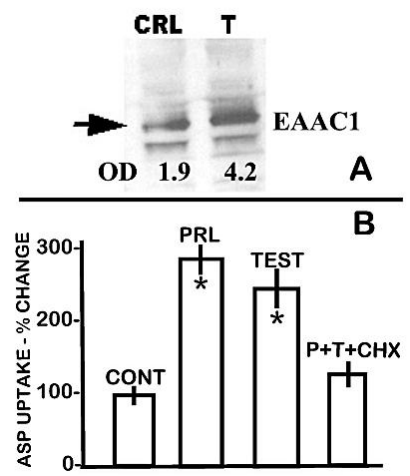
Effect of testosterone on EAAC1. **A. **Freshly prepared rat ventral VP epithelial cells were incubated for 3 hours in HBSS medium that contained 1 nM testosterone or vehicle (control). Loading gel (not shown) showed no loading differences between the control and testosterone samples. **B. **Effects of prolactin and testosterone on high-affinity aspartate transport of rat ventral prostate. The cells were harvested from donor animals that were castrated and bromocryptine-treated to reduce endogenous levels of hormones. CHX= 30 uM cyclohexamide; PRL= 1 nM; TEST= 10 nM. * P < 0.05 vs control.

An earlier report [6] demonstrated that testosterone increased the high-affinity transport of aspartate in the rat ventral prostate cells. Therefore we surmised that, if EAAC1 is the high-affinity transporter in prostate cells, testosterone treatment should be expected to increase the expression of EAAC1 in these cells. Figure 3 shows that exposure of the ventral prostate cells to testosterone results in a >100% increase in the level of EAAC1 transporter. This correlates with our earlier study of testosterone stimulation of high-affinity L-aspartate uptake in the prostate cells.

**Figure 4:**
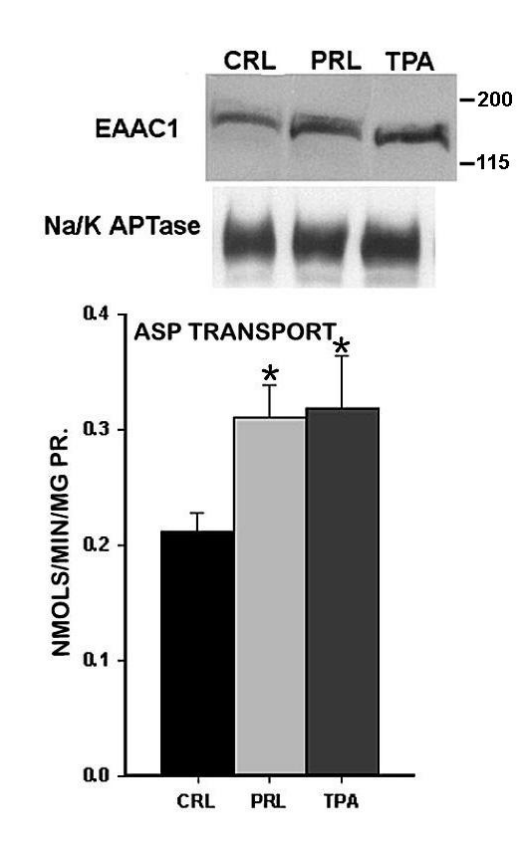
Effect of PRL and TPA on membrane-associated EAAC1 level and on L-aspartate uptake by normal rat ventral prostate cells. Prolactin (PRL) concentration was 1 nM and TPA concentration was 0.1 μg/ml for 3 hr. *= p < 0.05 compared to control (CRL).

In addition to testosterone, prolactin also regulates citrate production and key metabolic genes, including mAAT gene expression [[3], for review]; which caused us to consider the possibility that prolactin might also be important in the regulation of aspartate transport. Therefore, the effect of prolactin was determined, along with testosterone, on the high-affinity L-aspartate transport in the rat ventral prostate cells. In this study, the donor rats were castrated and bromocryptine-treated to minimize effects of endogenously produced hormones. The results (figure 3) show that prolactin and testosterone significantly increase the high-affinity transport of aspartate by 180% and 140%, respectively. Moreover, treatment with cyclohexamide attenuates the stimulatory effect of prolactin and testosterone; thereby indicating the dependency of the hormonal effects on gene expression. The effects of testosterone corroborate our earlier report [6]. Based on this observation, we determined the effect of prolactin on the expression of EAAC1 and correspondingly on aspartate transport in normal rat ventral prostate epithelial cells. Figure 4 shows that prolactin treatment increases the level of EAAC1; and correspondingly increases the uptake of aspartate. It is important to note that the level of hormonal stimulation is less than shown in figure 3, which is a reflection of the endogenous hormonal effect in the normal animals [4]. Previous studies have shown that prolactin regulation of metabolic genes in prostate cells is mediated via direct stimulation of the PKC signalling pathway and is mimicked by phorbol ester [3]. For this reason we also determined the effects of TPA (12-O-tetradecanoylphorbol-13-acetate). The stimulation of EAAC1 and aspartate transport by TPA as well as by prolactin is indicative of the involvement of PKC in the regulation of the EAAC1 gene (which we are now studying). These results further corroborate the relationship between EAAC1 and high-affinity L-aspartate transport in prostate cells, and also establish that prolactin as well as testosterone is involved in the hormonal regulation of EAAC1.

**Figure 5:**
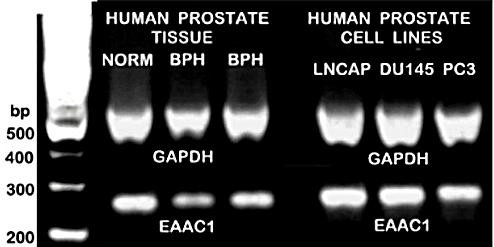
The expression of EAAC1 in human prostate glandular tissue and in human prostate cell lines. BPH=benign prostatic hyperplasia.

We were fortunate to obtain some archived human prostate tissue samples (courtesy of Dr. John Kurhanewicz, UCSF), which we analyzed for EAAC1 expression. At the same time we determined the expression of EAAC1 in LNCaP, DU-145, and PC-3 cells. Figure 5 shows that EAAC1 is expressed in human normal and hyperplastic glandular tissue as well as in the three malignant cell lines. Thus EAAC1 expression seemingly is ubiquitous in rat and human prostate cells.

## Discussion

**Figure 6:**
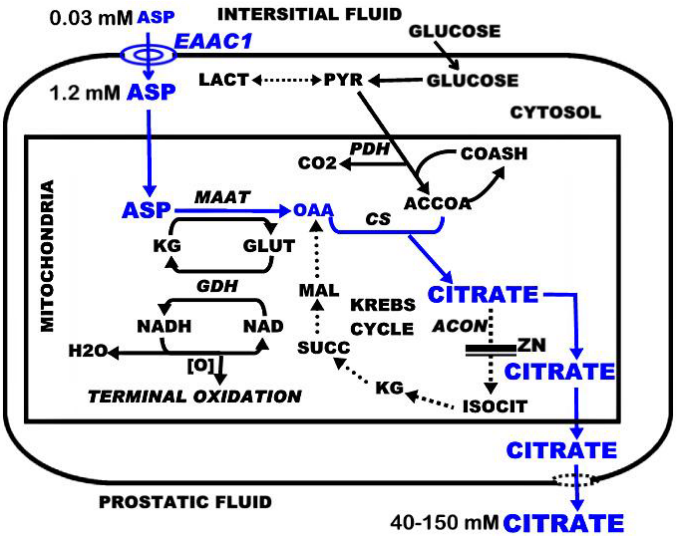
The role of aspartate and aspartate transport in the pathway of prostate citrate production. Aspartate via mAAT provides the four-carbon source of oxalacetate that condenses with acetyl coA for citrate synthesis. The oxidation of citrate via the Krebs cycle is prevented by the inhibition of m-aconitase by zinc. The utilized aspartate is replenished by the transport of aspartate from interstitial fluid by the EAAC1 aspartate transporter. Note the extremely high concentration of citrate in prostatic fluid secretion that requires a substantial and continuous supply of aspartate. mAAT = mitochondrial aspartate aminotransferase; GDH = glutamic dehydrogenase; CS = citrate synthase; ACON = mitochondrial aconitase; PDH = pyruvate dehydrogenase.

The present studies establish that alterations in the expression and level of EAAC1 transporter by siRNA knock-down or by hormonal stimulation are consistently accompanied by corresponding alterations in the high-affinity transport of aspartate. These studies in concert with our earlier reports [5-7] establish that EAAC1 is the functional transporter that is responsible for this high-affinity L-aspartate transport in rat and human prostate cells. This provides the physiological function for cellular accumulation of aspartate from circulation as is needed for their specialized function of net production of high levels of citrate. In these cells, aspartate is an essential amino acid that must be derived from circulation. The aspartate transporter Km ~10 uM aspartate (compared to 30 uM aspartate concentration in plasma) and other kinetic properties described earlier [5-7] provide for aspartate uptake and cellular accumulation against the existing 40:1 concentration gradient (intracellular: interstitial fluid) across the cell membrane. In addition, in the presence of a plasma concentration of 25 uM glutamate [10], the high affinity uptake of aspartate still occurs at greater than 50% of its maximal rate [6]. The enhancement of the high-affinity L-aspartate transport by the trans-stimulatory effect of high intracellular citrate provides additional effectiveness of the transporter for aspartate uptake by the prostate cells [7]. The importance of aspartate and EAAC1 as the high-affinity L-aspartate transporter in the unique function and citrate-related intermediary metabolism of prostate cells is depicted in figure 6.

The present study provides the first report of EAAC1 expression in prostate cells. More importantly, this report coupled to earlier studies [5-7] provides (to the best of our knowledge) the first definitive identification of EAAC1 as a preferential functional L-aspartate transporter. The operation of EAAC1 as a high-affinity L-aspartate transporter in prostate cells differs from its dominant focus as a glutamate transporter in excitatory cells. As such, the kinetic properties of EAAC1 as a high-affinity L-aspartate transporter have not been established for other mammalian cells. King et al [11] reported that L-aspartate uptake in heart myocytes is mediated by a high affinity sodium-dependent transporter (Km ~7 uM aspartate), and suggested that the transport might be associated with the expression in heart of EAAC1. Similarly, mammary cells reportedly express an X_AG_^- ^transporter that exhibits a significantly higher affinity for aspartate (Km = 32 uM) than for glutamate (Km = 112 uM); thereby potentially demonstrating a selectivity for aspartate transport [12]. In an analogous situation, Besson et al [13] reported that the nervous tissue-specific Drosophila EAAT2 preferentially functions as a Na+-dependent high-affinity aspartate transporter

The in situ operation and preference of plasma membrane-associated EAAC1 either as a high-affinity glutamate transporter or as a high-affinity aspartate transporter is determined by the intracellular and extracellular environment of the cell, and by the functional/metabolic activities of the cell. Unlike prostate cells, most mammalian cells synthesize aspartate and have no requirement for the extraction of aspartate from circulation. Moreover, glial cells and central neurons operate in the environment of cerebrospinal fluid and localized synaptic junctions that have a much different milieu composition than the common interstitial fluid environment of most cells. Even among cells that are exposed to the same interstitial fluid environment, the activity of plasma membrane transporters can differ as a result of differences in the intracellular activity and composition. This is particularly illustrated by the pronounced trans-stimulatory effect (11-fold increase in aspartate transport) of the high intracellular citrate concentration that exists in prostate cells but not in other mammalian cells [7]. Therefore, it is not surprising that EAAC1 shows variability in different cells in its functional preference for aspartate transport or for glutamate transport.

The effect of testosterone and prolactin on EAAC1 and high affinity L-aspartate transport has two important implications. As already discussed, the hormonal up-regulation of EAAC1 and corresponding increase in aspartate transport provide evidence that EAAC1 is the functional high-affinity L-aspartate transporter. Another implication relates to the regulation of the major prostate function of citrate production and secretion into prostatic fluid. Both hormones regulate (increase) prostate citrate production. They do so by regulation of gene expression of key regulatory enzymes in the pathway of citrate metabolism; pyruvate dehydrogenase, mAAT and m-aconitase [3]. The present report adds the EAAC1 transporter gene (*SLC1A1*) expression as a key step in the pathway to net citrate production. This is consistent with the regulation of mAAT gene expression; so that the aspartate substrate does not become limiting for the enzyme reaction in the direction of OAA production for citrate synthesis (figure 6). We have shown that prolactin regulation of mAAT gene expression is mediated via the diacylglycerol→ PKCepsilon → AP-1 signaling pathway (3,14,15). The stimulation of EAAC1 expression by both prolactin and TPA suggests that this PKC pathway is involved in regulation of the *SLC1A1 *gene, which we are now studying. Millar and Shennan [12] reported that treating lactating rats with bromocryptine reduced D-aspartate uptake by mammary tissue explants and suggested that the transport of anionic amino acids by the rat mammary gland is regulated by prolactin. It is of interest that phorbol ester activation of select PKC isoforms in some cells causes increased association of subcellular EAAC1 with the plasma membrane; which increases glutamate transport [16-18]. Whether or not such a mechanism applies to EAAC1 and aspartate transport in prostate cells needs to be investigated.

## Conclusion

Aspartate uptake and utilization are essential requirements for the unique function of production and secretion of extraordinarily high levels of citrate by prostate epithelial cells. To achieve this, these cells posses a high-affinity L-aspartate transporter that serves to extract aspartate from circulation. We now show that EAAC1 is expressed in human and rat prostate cells and functions as the high-affinity L-aspartate transporter in prostate cells. The expression of EAAC1 and the corresponding high-affinity transport of aspartate are up-regulated by testosterone and by prolactin, which correlates with the hormonal regulation of prostate citrate production. EAAC1 provides the high-affinity transporter for prostate cell uptake and accumulation of aspartate from circulation, which provides the source of OAA that is essential for the synthesis and secretion of extraordinarily high levels of citrate. The identification of EAAC1 as the aspartate transporter now permits studies of the *SLC1A1 *gene for identification of the mechanism of testosterone and prolactin regulation, and permits studies of the kinetic relationships and mechanisms of preferential and enhanced aspartate transport by EAAC1 in prostate and other cells. Moreover, the identification of EAAC1 in human normal and hyperplastic glandular tissue raises the need to determine its expression in prostate adenocarcinomatous glands; and such a study is in progress.

## Methods

### Preparation of prostate cells and hormone treatment

The animals used in these studies were maintained in accordance with the NIH guidelines for the care and use of laboratory animals. In experiments that involved the effects of hormone, 8–10 animals were castrated over night to reduce endogenous androgen levels or treated with bromocryptine (Sigma Aldrich) to reduce the endogenous level of prolactin as previously described [14,19]. Twenty-four hours after castration or bromocryptine treatment, prostate tissue was resected and isolated ventral prostate cells prepared by collagenase digestion as previously described [14]. Cells were then incubated with testosterone (10^-8 ^M), prolactin (10^-9 ^M) or TPA for 3 hours based on our previous studies [6,15]. Cells were collected, washed and used for L-aspartate uptake assay or Western blot analysis. Controls were treated with the appropriate vehicle. Each experiment was repeated at lease two times with triplicate samples for each data point. Data are shown as mean and s.e.m.

### High affinity L-aspartate uptake assay

The preparation of freshly isolated rat ventral prostate epithelial cells and the conditions for high-affinity L-aspartate transport are detailed in our previous reports [5-7]. Transport was conducted by adding 50 ul of cell suspension (10^6 ^cells) to 1.0 ml Tris-Na+ medium containing ^3^H-labelled L-aspartate at a final concentration of 30 uM which is the plasma concentration of L-aspartate. Uptake was conducted for either 1 min and 5 min or 1 min and 10 min at 37°C to ensure that the 1 min uptake was during a linear phase of uptake. After the uptake incubation the cells were rapidly washed, collected on filters and counted in a liquid scintillation counter. All assays were run in triplicate and the mean +/- SEM determined for t-test statistical analyses.

### Cell culture and siRNA treatment

PC-3 cells were obtained from the American Type Culture Collection (ATCC, Manassas, VA). The cells were cultured in RMPI 1640 medium supplemented with 10% fetal bovine serum, 50 units/ml penicillin and 50 mg/ml streptomycin as previously described [14]. Cells were treated in 6 well plates with siGENOME EAAC1 Smart Pool siRNA from Dharmacon RNA Technologies. Initial experiments showed that Smart Pool sequences 3 and 5 resulted in the most knock-down of EAAC1 expression; therefore subsequent experiments were carried out using sequence 5. The sequence of siRNAs 3 and 5 were 5'-GGAAGAUCAUAGAAGUUGAUU-3' and 5'-GCUGAUAUAUUUCAUAGUCUU-3' respectively. Cells were transfected with 20 nM/well siRNA using TransIT-TKO (Mirus) reagent according to the manufacture's protocol. After 72 hrs cells were collected washed and lysates prepared for Western blot analysis or cells were used for assay of high-affinity L-aspartate transport as described above for rat ventral prostate cells. Analysis of EAAT1 expression showed that the siRNA treatment was specific for EAAC1.

### Western Blot for aspartate transporters

The membrane fraction was prepared from whole cell lysates as we have previously described [14]. Extracted membrane proteins were separated by SDS-PAGE, transferred to nitrocellulose membrane and probed using specific antibodies for EAAC1 and EAAT1 purchased from Alpha Diagnostics International. Membranes were incubated with a 1 to 1000 dilution of primary antibody followed by incubation with anti-rabbit IgG horseradish preoxidase linked secondary antibody. Protein bound antibody was detected using ECL detection reagents from Amersham Cooperation. Membrane Na/K ATPase used as a control for loading membrane proteins was detected using an antibody from Sigma Aldrich. Western blots were quantified by scanning the film and analyzing the intensity of bands using SigmaScan software.

### RT-PCR for aspartate transporters

Total RNA was extracted from rat ventral prostate and brain tissue using TRIzol Reagent (Invitrogen, Carlsbad CA). Two μg of total RNA were reverse transcribed using a TaqMan Gold RT-PCR Kit (Applied Biosystems). Final reaction concentrations were as follows: 2.5 μmol/L random hexamer, 1.25 U/μL Multiscribe reverse transcriptase, 1X TaqMan buffer, 0.4 U/μL RNase inhibitor, 5.5 mmol/L MgCl2, and 500 μmol/L each dNTP. Reverse transcription was conducted at 25°C for 10 min, 48°C for 30 min, and 95°C for 5 min. An aliquot of 2.5 μl of the synthesized cDNA served as the PCR template. The conditions for PCR amplification were as follows: 94°C for 3 min; 40 cycles of 94°C for 1 min, 57°C for 1 min, 72°C for 1 min. Primers for EAAC1, EAAT1, EAAT2 and EAAT4 were: 5'-GTCATTCTGCCAATTAT-3' and 5'-GATGCCGTCTGACAG-3'; 5'-GATTTGCCCTCCGACCGTAT-3' and 5'-ATGTTTCCAATCACGAAGCC-3'; CTCACTGACTGTGTTTGGTG-3' and 5'-CTAAGACATTCATCCCGTCC-3'; 5'-CGAGTGGTAACAAGGACGAT-3' and 5'-GTTCCGTGTGACAAGG-3', respectively.

## Authors' contributions

RB and LC conceived the ideas and experimental design, oversaw the experiments and data analyses, prepared the manuscript. JZ maintained and harvested the cells in culture, set up the animal experiments and tissue cell preparation, performed the high-affinity aspartate uptake studies. ZU carried out the molecular genetic studies, performed the RT-PCR analysis, developed and checked primers. All authors read and approved the final manuscript.
